# Algicidal lactones from the marine *Roseobacter* clade bacterium *Ruegeria pomeroyi*

**DOI:** 10.3762/bjoc.8.106

**Published:** 2012-06-25

**Authors:** Ramona Riclea, Julia Gleitzmann, Hilke Bruns, Corina Junker, Barbara Schulz, Jeroen S Dickschat

**Affiliations:** 1 Institut für Organische Chemie, Technische Universität Braunschweig, Hagenring 30, D-38106 Braunschweig, Germany; 2 Institut für Mikrobiologie, Technische Universität Braunschweig, Spielmannstraße 7, D-38106 Braunschweig, Germany

**Keywords:** bacteria-algae symbiosis, lactones, *Roseobacter*, synthesis, volatiles

## Abstract

Volatiles released by the marine *Roseobacter* clade bacterium *Rugeria pomeroyi* were collected by use of a closed-loop stripping headspace apparatus (CLSA) and analysed by GC–MS. Several lactones were found for which structural proposals were derived from their mass spectra and unambiguously verified by the synthesis of reference compounds. An enantioselective synthesis of two exemplary lactones was performed to establish the enantiomeric compositions of the natural products by enantioselective GC–MS analyses. The lactones were subjected to biotests to investigate their activity against several bacteria, fungi, and algae. A specific algicidal activity was observed that may be important in the interaction between the bacteria and their algal hosts in fading algal blooms.

## Introduction

**Figure 1:**
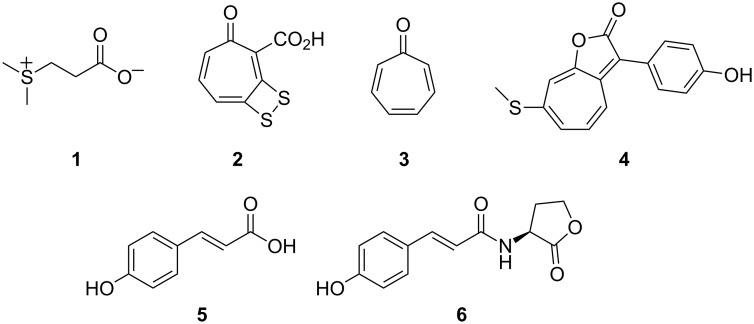
Important metabolites in the interaction of bacteria from the *Roseobacter* clade with marine algae.

Bacteria of the *Roseobacter* clade form one of the most abundant lineages of marine bacteria that occur globally in marine ecosystems from polar to tropical regions [1–2]. They are present in costal and open ocean environments, in surface waters and in the water column; are found as algal symbionts [3–4] or associated with molluscs [5]; and can form biofilms [6]. Particularly interesting from an ecological point of view is their association with marine algae, such as dinoflagellates and coccolithophores, which produce large amounts of the sulfur metabolite dimethylsulfoniopropionate (**1**, DMSP, Figure 1). DMSP is used as an osmolyte and cryoprotectant by marine phytoplankton, various macroalgae, and also a few angiosperms, and is produced at an estimated annual rate of 1000 Tg (10^15^ g) [7]. The microalgal phytoplankton frequently forms massive blooms, which can even be observed by satellites from space [8], sometimes covering large areas of >10^5^ km^2^ and containing more than 10^6^ cells mL^−1^. During these blooms bacteria from the *Roseobacter* clade have been observed as the predominant prokaryotic species accounting for more than half of the total bacterial community [3–4]. DMSP is also an attractant for *Ruegeria* sp. TM1040 and causes flagella-mediated chemotactic behaviour [9], suggesting an important role of DMSP in the symbiosis between the algae and bacteria. Upon lysis of ageing blooms by viruses, or cell disruption by grazing, the intracellular DMSP is released, making the dissolved DMSP available for bacterial degradation to methanethiol (MeSH) [10] or dimethyl sulfide (DMS) [11–15]. The bacterial production of DMS is important for the global sulfur cycle [16–17] and the planet’s climate [18–19], while the alternative DMSP degradation product MeSH controls the bioavailability of metal ions by the formation of metal–MeSH complexes [7] and can be used for the biosynthesis of various sulfur-containing secondary metabolites [20].

A sulfur-containing metabolite, for which the direct sulfur precursor has not been determined yet, is the antibiotic tropodithietic acid (TDA, **2**), which may have an important function in mutualistic symbioses of *P. gallaeciensis* and marine algae by protecting the algal host from pathogenic bacteria in emerging blooms. In ageing blooms *p*-coumaric acid (**5**) is released from lysing algal cells as a lignin breakdown product. This compound causes a switch in *P. gallaeciensis* from exhibiting mutualistic to pathogenic properties mediated by the algicidal roseobacticides, which are only produced upon induction by **5** [21–22]. Roseobacticide A (**4**) was suggested to arise from tropone (**3**), *p*-hydroxyphenylacetic acid, which is potentially formed from **5**, and MeSH [21]. In addition, **5** can be taken up and used by some bacteria, including *R. pomeroyi* for the biosynthesis of the autoinducer *N*-coumaroyl-L-homoserine lactone (**6**) [23], but it is unknown whether **6** or any other molecule plays a regulatory function for the genetic activation of the biosynthesis of **4**, or whether formation of the roseobacticides is just activated because the required building block is available from **5**.

All of these recently obtained insights support a strong interaction between marine algae and bacteria of the *Roseobacter* clade, which is mediated by small and diffusible molecules. Herein we describe the identification and synthesis of volatile lactones from *R. pomeroyi* and their specific algicidal activity, which may also play a role in the interaction between the *Roseobacter* clade bacteria and their algal hosts in fading blooms.

## Results and Discussion

### Analysis of volatiles released by *Ruegeria pomeroyi*

**Figure 2:**
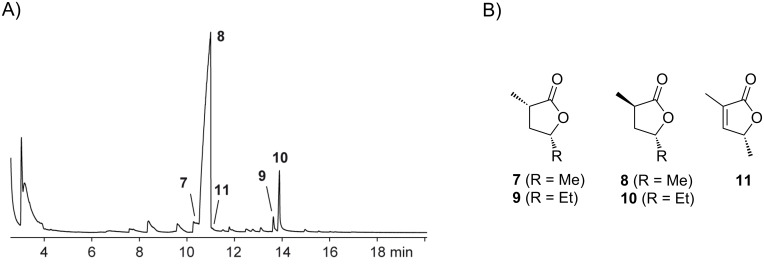
(A) Total ion chromatogram of a headspace extract from *R. pomeroyi*, (B) structures of lactones released by *R. pomeroyi*.

The volatiles emitted by agar plate cultures of *Ruegeria pomeroyi* DSS-3 grown on ½ YTSS medium were collected on charcoal by using a closed-loop stripping apparatus (CLSA) [24]. After a collection time of about one day the adsorbed compounds were eluted with analytically pure dichloromethane, and the obtained extracts were analysed by GC–MS. A representative chromatogram of a headspace extract of *R. pomeroyi* is shown in Figure 2A.

**Figure 3:**
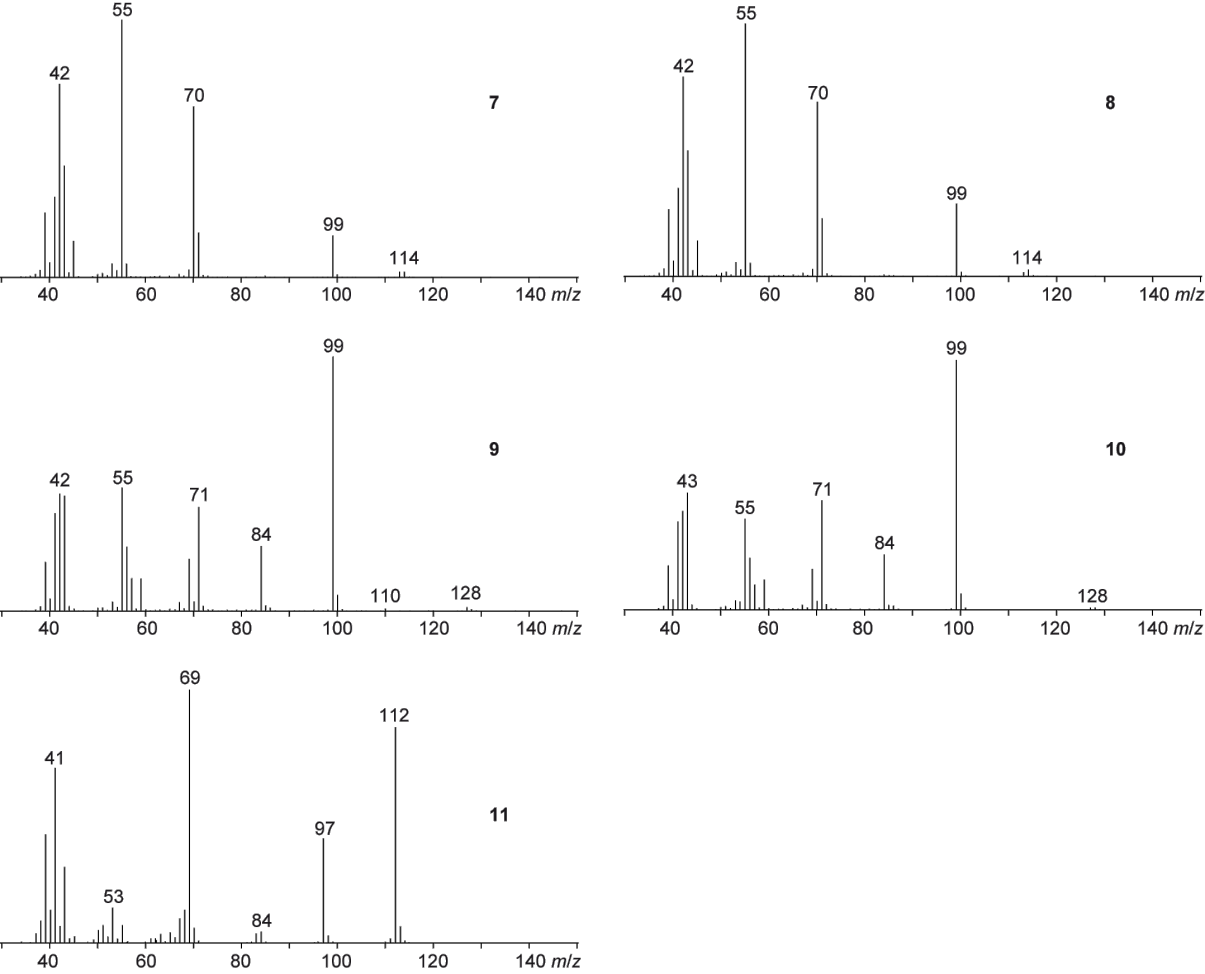
Mass spectra of the compounds **7**–**11** emitted by *R. pomeroyi*.

The sulfur volatiles dimethyl disulfide, dimethyl trisulfide, and *S*-methyl methanethiosulfonate, previously reported from several other bacteria of the *Roseobacter* clade and also from various other species [25], were readily identified according to their mass spectra and by comparison with synthetic standards. However, the volatiles **7**–**11**, including the main compound **8**, could not immediately be identified by their mass spectra alone. The mass spectrum of **8** (Figure 3) showed strong similarities to the mass spectrum of 2-methylpentan-4-olide, which is included in our electronic mass-spectra libraries [26–27]. Compound **7**, which is released only in small amounts, showed an almost identical mass spectrum, suggesting the volatiles **7** and **8** to be the *cis*- and *trans*-diastereoisomers of 2-methylpentan-4-olide, but it was impossible to assign the structure of a distinct diastereomer. The compounds **9** and **10** also showed highly similar mass spectra and both a molecular ion at *m*/*z* = 128. Due to the increase by 14 amu compared to the molecular ions of **7** and **8**, the volatiles **9** and **10** were assumed to represent higher homologues by the addition of one methylene unit. The base peak at *m*/*z* = 99 furthermore supported the structures of methylated butanolides, leading to the structural suggestions of *cis*- and *trans*-2-methylhexan-4-olide. The alternative structures of *cis*- and *trans*-2-ethylpentan-4-olide seemed less likely since these lactones were assumed to undergo a McLafferty rearrangement that should result in significant fragment ions at *m*/*z* = 100 by the neutral loss of ethene. Finally, compound **11** exhibited a mass spectrum with a molecular ion at *m*/*z* = 112, which, together with the fragment ion at *m*/*z* = 97, suggested the structure of a dimethylbutenolide. Compound **11** may be the precursor of, or derived from, **7** and **8**, resulting in the proposed structure of 2-methylpent-2-en-4-olide.

**Scheme 1:**
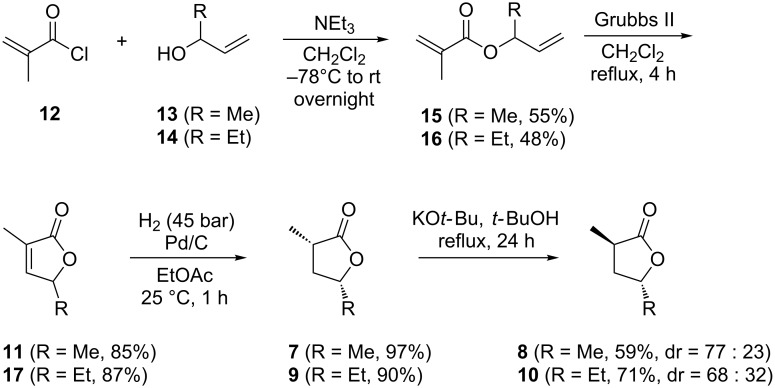
Synthesis of compounds **7**–**11**. For these target structures the relative configurations are shown.

To prove the structural suggestions unambiguously, syntheses of reference compounds were carried out (Scheme 1). Methacryloyl chloride (**12**) was esterified with but-3-en-2-ol (**13**) in the presence of triethylamine to yield but-3-en-2-yl methacrylate (**15**). Ring-closing metathesis with Grubbs catalyst of the second generation gave 2-methylpent-2-en-4-olide (**11**) that upon catalytic hydrogenation yielded *cis*-2-methylpentan-4-olide (**7**) as a single diastereomer, as reported previously [28]. Under prolonged treatment with KO*t*-Bu in *t*-BuOH under reflux, partial isomerisation to *trans*-2-methylpentan-4-olide (**8**) was achieved. Longer reaction times did not result in higher yields of the *trans* isomer, but instead in loss of material due to decomposition, and therefore the isomerisation was stopped after one day. By using the same approach starting from **12** and pent-1-en-3-ol (**14**) pure *cis*-2-methylhexan-4-olide (**9**) was obtained by esterification to pent-1-en-3-yl methacrylate (**16**), ring-closing metathesis to 2-methylhex-2-en-4-olide (**17**), and catalytic hydrogenation. The isomerisation of **9** with KO*t*-Bu in *t*-BuOH again provided a mixture of **9** and its diastereomer *trans*-2-methylhexan-4-olide (**10**). Comparison of GC retention times and mass spectra of the synthetic material to those of the natural compounds revealed that the first-eluting minor diastereomer of 2-methylpentan-4-olide emitted by *R. pomeroyi* is the *cis*-diastereomer **7** and the main compound is the *trans*-diastereomer **8**. Accordingly, the structures of the *cis*- and *trans*-diastereomers **9** and **10** were assigned to the first- and the second-eluting diastereomers of 2-methylhexan-4-olide, respectively. Furthermore, the trace compound **11**, found in the headspace extract, was identical to the synthetic intermediate obtained en route to **7** and **8**.

**Scheme 2:**
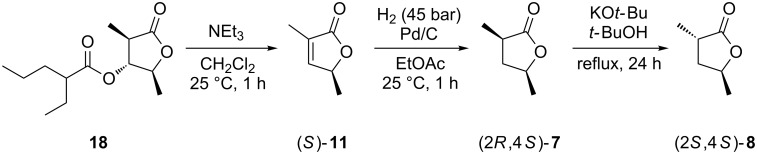
Enantioselective synthesis of (2*R*,4*S*)-**7** and (2*S*,4*S*)-**8**.

**Figure 4:**
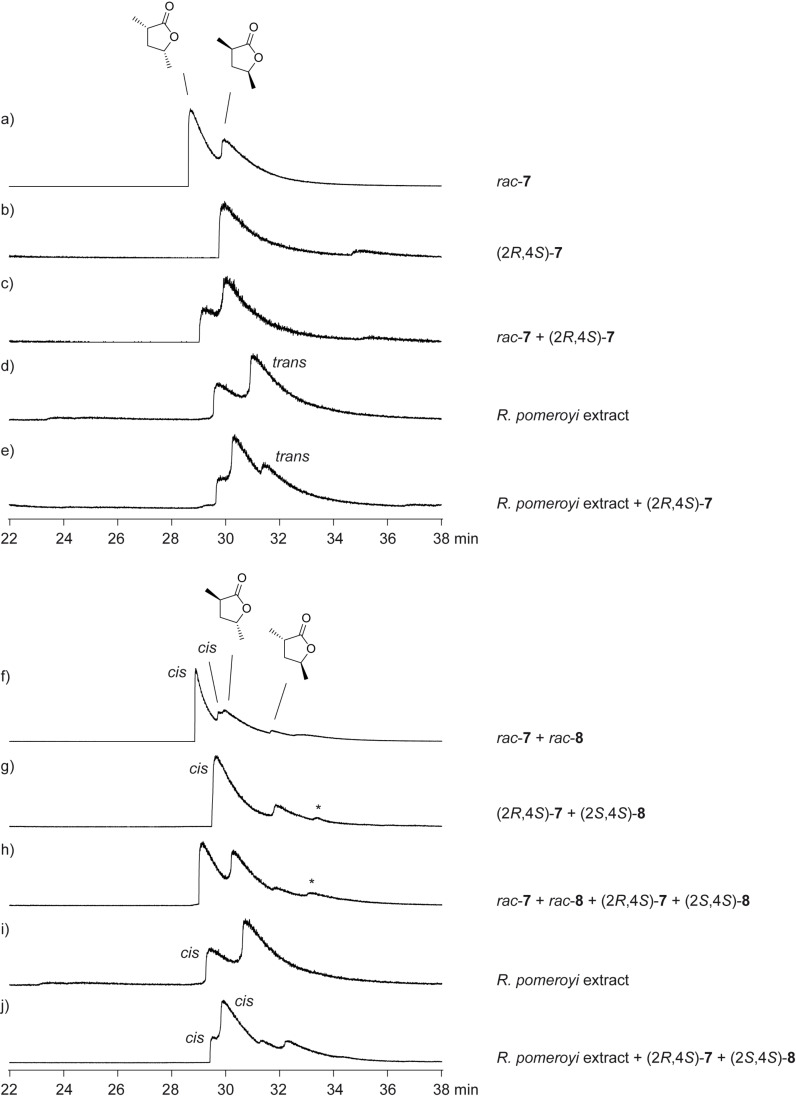
Enantioselective GC analyses for the assignment of the enantiomeric compositions of natural (2*S*,4*R*)-**7** and (2*R*,4*R*)-**8** from *R. pomeroyi*.

To determine the enantiomeric compositions of the lactones from *R. pomeroyi*, an enantioselective synthesis of **7** and **8** was carried out (Scheme 2). For this purpose the lactone (2*S*,3*R*,4*R*)-2,4-dimethyl-5-oxotetrahydrofuran-3-yl 2-ethylpentanoate (**18**), a derivative of the antimycin degradation product blastmycinone, which has recently been obtained in an enantioselective synthesis in our laboratory [29], was used as a starting material. The elimination of 2-ethylpentanoic acid was achieved by treatment with triethylamine to yield (*S*)-**11**. As described above for the racemic compounds, catalytic hydrogenation afforded (2*R*,4*S*)-**7**, which was isomerised to (2*S*,4*S*)-**8**. Enantioselective GC-analyses clearly demonstrated that the lactones from *R. pomeroyi* have the opposite absolute configurations as these synthetic lactones (Figure 4). Therefore, the lactones from *R. pomeroyi* are identified as (2*S*,4*R*)-**7** and (2*R*,4*R*)-**8**. The bacterial headspace extracts did not contain sufficient amounts of **11** for elucidation of its absolute configuration by using synthetic racemic and (*S*)-**11**. However, the absolute configurations of the other lactones are most likely related to those of **7** and **8**, leading to the suggested structures of (2*S*,4*R*)-**9**, (2*R*,4*R*)-**10**, and (*R*)-**11**. As can be seen in an accompanying paper in this Thematic Series by Francke and co-workers on the synthesis and absolute configurations of iridomyrmecins from the parasitoid wasp *Alloxysta victrix*, the expected stereochemical relationships between structurally similar natural products from one species are not always met [30], and therefore, these suggestions should be taken with care.

To investigate the possible biological function of the lactones emitted by *R. pomeroyi* an agar diffusion assay with the synthetic compounds was carried out (Table 1). Since the headspace extracts from *R. pomeroyi* contained mixtures of the diastereomers **7**/**8** and **9**/**10**, respectively, these compounds were also tested as diastereomeric mixtures as obtained in the isomerisation procedures with **7** and **9**. Tests were performed with bacteria, including the Gram-negative *Escherichia coli* and the Gram-positive *Bacillus megaterium*, fungi, represented by the basidiomycete *Microbotryum violaceum* and the ascomycete *Botrytis cinerea*, and the fresh water alga *Chlorella fusca*. These microorganisms were chosen because they are nonpathogenic and are accurate initial test organisms for antibacterial, antifungal, and antialgal/herbicidal activities. All the lactones specifically showed partial inhibition of the alga *C. fusca*, but no activity against the bacteria and fungi.

**Table 1:** Agar diffusion assay with the lactones released by *Ruegeria pomeroyi*.^a^

Compound	*E. coli*^b^	*B. megaterium*^c^	*M. violaceum*^d^	*B. cinerea*^e^	*C. fusca*^f^

**7**/**8**^g^	0	0	0	0	6 (pi)
**9**/**10**^g^	0	0	0	0	6 (pi)
**11**	0	0	0	0	7 (pi)
penicillin	nt	18	0	nt	0
tetracycline	nt	18	0	nt	10 (pi)
nystatin	nt	0	20	nt	0
actidione	nt	0	50	nt	35
MeOH	0	0	0	0	0

^a^Radius of inhibition zones in mm, pi = partial inhibition, nt = not tested; ^b^*Escherichia coli* K12; ^c^*Bacillus megaterium*; ^d^*Microbotryum violaceum*; ^e^*Botrytis cinerea*; ^f^*Chlorella fusca*; ^g^diastereomeric mixtures as obtained in the isomerisations of **7** and **9** were used.

## Conclusion

More and more data are accumulated demonstrating that bacteria of the marine *Roseobacter* clade produce bioactive secondary metabolites. In the present work, we have identified five lactones in the volatile fraction of *R. pomeroyi*. The structures of these lactones have been unambiguously assigned by comparison to synthetic standards. In agar diffusion assays the synthetic lactones showed specific activity against algae, but not against bacteria or fungi, suggesting that the lactones may have an ecological function in the interaction between the bacteria and the algae in fading algal blooms, similar to the recently described roseobacticides from *P. gallaeciensis*, which are active against *Emiliana huxleyi*. In the present work we have performed initial tests to investigate the bioactivity of the *Ruegeria* lactones against bacteria, fungi, and the fresh water alga *Chlorella fusca*. Further tests will have to be performed with marine algae, including *E. huxleyi* and related species, to investigate the significance of these findings within the ecological context of the bacterial lactone producers.

## Experimental

**Strains, media, and growth conditions.** *Ruegeria pomeroyi* DSS-3 was grown in ½ YTSS liquid medium (2 g L^−1^ yeast extract, 1.25 g L^−1^ tryptone, 20 g L^−1^ sea salts (Sigma-Aldrich)) at 28 °C. After full growth of the culture (ca. 3 d), an agar plate with YTSS medium was inoculated by plating of 100 μL of liquid culture. Plates were incubated for three days and then subjected to headspace analysis.

**Collection of volatiles.** The volatiles released by the *R. pomeroyi* agar plate cultures were collected by use of a closed-loop stripping apparatus (CLSA) as described previously [24]. The headspace extracts were immediately analysed by GC–MS and stored at −80 °C.

**GC–MS.** GC–MS analyses were carried out with a HP7890A gas chromatograph connected to a HP5975C mass-selective detector. The GC was equipped with a HP-5 MS fused silica capillary column (30 m × 0.22 mm i.d., 0.25 μm film, Hewlett-Packard, Wilmington, USA) or with a hydrodex-6-TBDMS fused silica capillary column (50 m, 0.25 mm i.d., 0.25 μm film, Macherey-Nagel) for enantioselective GC analyses. Conditions were as follows: inlet pressure: 67 kPa, He 23.3 mL min^−1^; injection volume: 1 μL; injector: 250 °C; transfer line: 300 °C; electron energy: 70 eV; carrier gas (He): 1.2 mL min^−1^. The GC was programmed as follows: standard GC analyses: 50 °C (5 min isothermic), increasing at 5 °C min^−1^ to 320 °C; enantioselective GC analyses: 35 °C, increasing at 0.75 °C min^−1^ to 65 °C, followed by 20 °C min^−1^ to 220 °C. Retention indices were determined from a homologous series of *n*-alkanes (C_8_–C_32_). For compound identification commercially available mass-spectrum libraries were used [26–27].

**General synthetic methods:** The syntheses of the reference compounds **7**/**8** and **9**/**10** were performed by using the same route. In the following paragraphs general procedures are given in which the molar ratios of the starting materials are given in equivalents (equiv). Concentrations refer to the transformed starting material, which was set to 1.0 equiv in the appropriate solvents. All reactions were performed in flame-dried glassware in a nitrogen atmosphere. Solvents were dried according to standard methods. All chemicals were obtained from commercial suppliers (Sigma-Aldrich or Acros) and used without further purification.

**General procedure for the preparation of methacrylate esters:** A 0.3 M solution of the appropriate alcohol **13** or **14** (1.0 equiv) and NEt_3_ (1.38 equiv) in dry dichloromethane was cooled to 0 °C followed by the slow addition of methacryloyl chloride (**12**, 1.0 equiv). The mixture was stirred at room temperature overnight and then quenched by the addition of an equal volume of 1 N HCl. The layers were separated and the aqueous layer was extracted three times with dichloromethane. The combined organic layers were washed with saturated aqueous NaHCO_3_ and brine, dried over MgSO_4_, and concentrated under reduced pressure. The residue was purified by column chromatography on silica gel (hexane/ethyl acetate 20:1) to yield the esters as colourless oils.

**But-3-en-2-yl methacrylate (15):** Yield: 0.92 g (6.6 mmol, 55%). TLC (hexane/ethyl acetate 20:1): *R*_f_ 0.50; GC (BPX-5): *I* = 909; ^1^H NMR (400 MHz, CDCl_3_) δ 6.11 (m, 1H, CH_2_), 5.86 (ddd, ^3^*J*_H,H_ = 5.8, 10.5, 16.3 Hz, 1H, CH), 5.54 (quin, ^2^*J*_H,H_ = ^4^*J*_H,H_ = 1.6 Hz, 1H, CH_2_), 5.43–5.35 (m, 1H, CH), 5.24 (dt, ^2^*J*_H,H_ = 1.2 Hz, ^3^*J*_H,H_ = 17.2 Hz, 1H, CH_2_), 5.12 (dt, ^2^*J*_H,H_ = 1.2 Hz, ^3^*J*_H,H_ = 10.5 Hz, 1H, CH_2_), 1.93 (dd, ^4^*J*_H,H_ = 1.1, 1.6 Hz, 3H, CH_3_), 1.34 (d, ^3^*J*_H,H_ = 6.5 Hz, ^1^*J*_C,H_ = 127.8 Hz, 3H, CH_3_); ^13^C NMR (100 MHz, CDCl_3_) δ 166.4 (CO), 137.7 (CH), 136.6 (C_q_), 125.4 (CH_2_), 115.5 (CH_2_), 77.1 (CH), 19.9 (CH_3_), 18.2 (CH_3_); MS (EI, 70 eV) *m*/*z* (%): 140 (<1) [M^+^], 111 (16), 95 (18), 69 (96), 55 (100), 53 (19), 43 (21), 41 (92), 40 (20); IR (ATR) 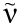: 2985 (w), 2933 (w), 2847 (w), 1731 (m), 1677 (m), 1450 (w), 1376 (w), 1344 (w), 1284 (m), 1242 (w), 1202 (m), 1181 (m), 1152 (s), 1110 (s), 1036 (s), 990 (m), 924 (m), 834 (w), 761 (w), 696 (w), 553 (w) cm^−1^; UV–vis λ_max_ (log ε): 240 (2.22) nm.

**Pent-1-en-3-yl methacrylate (16):** Yield: 3.0 g (19.4 mmol, 48%). TLC (hexane/ethyl acetate 10:1): *R*_f_ 0.29; GC (BPX-5): *I* = 988; ^1^H NMR (400 MHz, CDCl_3_) δ 6.11 (m, 1H, CH_2_), 5.81 (ddd, ^3^*J*_H,H_ = 17.0, 10.5, 6.5 Hz, 1H, CH), 5.54–5.52 (m, 1H, CH_2_), 5.25–5.19 (m, 2H, CH, CH_2_), 5.17–5.13 (m, 1H, CH_2_), 1.93 (d, ^4^*J*_H,H_ = 1.0 Hz, 3H, CH_3_), 1.69 (quintd, ^3^*J*_H,H_ = 7.3 Hz, ^4^*J*_H,H_ = 2.7 Hz, 2H, CH_2_), 0.90 (t, ^3^*J*_H,H_ = 7.4 Hz, ^1^*J*_C,H_ = 126.2 Hz, CH_3_); ^13^C NMR (100 MHz, CDCl_3_) δ 166.7 (CO), 136.6 (C_q_), 136.3 (CH), 125.1 (CH_2_), 116.5 (CH_2_), 76.0 (CH), 27.2 (CH_2_), 18.3 (CH_3_), 9.3 (CH_3_); MS (EI, 70 eV) *m*/*z* (%): 154 (<1) [M^+^], 125 (8), 111 (7), 109 (6), 69 (100), 67 (14), 41 (44), 39 (22); IR (ATR) 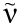: 3087 (w), 2696 (m), 2931 (m), 2879 (w), 1721 (s), 1640 (w), 1456 (w), 1381 (w), 1294 (w), 1261 (w), 1164 (m), 1078 (w), 1059 (w), 990 (w), 930 (m), 810 (w), 657 (w) cm^−1^; UV–vis: λ_max_ (log ε): 228 (2.83) nm.

**General procedure for the ring-closing metathesis to butenolides:** Grubbs catalyst of the second generation (0.05 equiv) was added to a solution of the ester **15** or **16** (1.0 equiv) in dry dichloromethane (0.05 M). The mixture was stirred under reflux for 5 d. The solvent was removed under reduced pressure and the residue was purified by column chromatography on silica gel (pentane/diethyl ether 3:1) to give the butenolides as colourless oils.

**2-Methylpent-2-en-4-olide (11):** Yield: 0.48 g (3.96 mmol, 85%). TLC (hexane/ethyl acetate 3:1): *R*_f_ 0.27; GC (BPX-5): *I* = 1017; ^1^H NMR (400 MHz, CDCl_3_) δ 7.02 (quint, ^3^*J*_H,H_ = ^4^*J*_H,H_ = 1.6 Hz, ^1^*J*_C,H_ = 171.3 Hz, 1H, CH), 5.01–4.94 (m, 1H, CH), 1.90 (dd, ^4^*J*_H,H_ = ^5^*J*_H,H_ = 1.6 Hz, ^1^*J*_C,H_ = 128.8 Hz, 3H, CH_3_), 1.39 (d, ^3^*J*_H,H_ = 6.8 Hz, ^1^*J*_C,H_ = 129.0 Hz, 3H, CH_3_); ^13^C NMR (100 MHz, CDCl_3_) δ 173.9 (CO), 149.6 (CH), 129.3 (C_q_), 77.1 (CH), 18.7 (CH_3_), 10.2 (CH_3_); MS (EI, 70 eV) *m*/*z* [%]: 112 (28), 98 (16), 84 (4), 69 (42), 52 (24), 41 (78), 39 (100); IR (ATR) 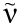: 3382 (br, w), 3084 (w), 2985 (w), 2933 (w), 1743 (s), 1659 (w), 1448 (w), 1376 (w), 1342 (w), 1321 (w), 1209 (w), 1188 (w), 1103 (m), 1082 (s), 1048 (m), 1028 (m), 997 (s), 929 (m), 866 (m), 763 (m), 610 (m), 574 (w), 540 (m) cm^−1^; UV–vis λ_max_ (log ε): 228 (2.70), 221 (2.03) nm. NMR spectroscopic data are in agreement with previously published data [31].

**2-Methylhex-2-en-4-olide (17):** Yield: 0.24 g (1.89 mmol, 97%). TLC (hexane/ethyl acetate 3:1): *R*_f_ 0.25; GC (BPX-5): *I* = 1113; ^1^H NMR (400 MHz, CDCl_3_) δ 7.02 (quint, ^3^*J*_H,H_ = ^4^*J*_H,H_ = 1.6 Hz, 1H, CH), 4.84–4.79 (m, 1H, CH), 1.89 (t, ^4^*J*_H,H_ = 1.8 Hz, 3H, CH_3_), 1.80–1.69 (m, 1H, CH_2_), 1.65 (dquin, ^2^*J*_H,H_ = 14.4 Hz, ^3^*J*_H,H_ = 7.2 Hz, 1H, CH_2_), 0.97 (t, ^3^*J*_H,H_ = 7.4 Hz, 3H, CH_3_); ^13^C NMR (100 MHz, CDCl_3_) δ 174.3 (CO), 148.4 (CH), 130.0 (C_q_), 82.0 (CH), 26.5 (CH_2_), 10.5 (CH_3_), 9.0 (CH_3_); MS (EI, 70 eV) *m*/*z* (%): 126 (41), 111 (28), 97 (100), 83 (3), 69 (64), 57 (17), 53 (6), 51 (5), 41 (56), 39 (35); IR (ATR) 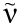: 2973 (w), 2932 (w), 2882 (w), 1744 (s), 1660 (w), 1459 (w), 1343 (w), 1281 (w), 1208 (w), 1086 (s), 1047 (m), 1022 (m), 959 (m), 856 (m), 786 (m), 761 (w), 612 (w), 555 (w) cm^−1^; UV–vis λ_max_ (log ε): 229 (2.83) nm. NMR spectroscopic data are in agreement with previously published data [32].

**General procedure for the catalytic hydrogenation of butenolides:** To a solution of the lactone **11** or **17** (1.0 equiv) in ethyl acetate (0.15 M) Pd on charcoal (10% Pd, 0.1 equiv) was added. The mixture was stirred in a hydrogen atmosphere (45 bar) for 1 h at 25 °C. The catalyst was removed by filtration, the solvent was evaporated under reduced pressure, and the residue was purified by column chromatography on silica gel (pentane/diethyl ether 3:1) to yield the butanolides as colourless oils.

***cis*-2-Methylpentan-4-olide (7):** Yield: 0.38 g (3.28 mmol, 97%). TLC (hexane/ethyl acetate 2:1): *R*_f_ 0.48; GC (BPX-5): *I* = 1005; ^1^H NMR (400 MHz, CDCl_3_) δ 4.40 (ddq, ^3^*J*_H,H_ = 5.4, 11.5, 6.1 Hz, 1H, CH), 2.66 (ddq, ^3^*J*_H,H_ = 8.5, 12.1, 7.1 Hz, 1H, CH), 2.49 (ddd, ^2^*J*_H,H_ = 12.4 Hz, ^3^*J*_H,H_ = 5.4, 8.5 Hz, 1H, CH_2_), 1.49 (dt, ^2^*J*_H,H_ = ^3^*J*_H,H_ = 12.3 Hz, ^3^*J*_H,H_ = 10.4 Hz, 1H, CH_2_), 1.39 (d, ^3^*J*_H,H_ = 6.1 Hz, 3H, CH_3_), 1.23 (d, ^3^*J*_H,H_ = 7.0 Hz, 3H, CH_3_); ^13^C NMR (100 MHz, CDCl_3_) δ 179.5 (CO), 74.9 (CH), 39.1 (CH_2_), 36.3 (CH), 20.9 (CH_3_), 15.1 (CH_3_); MS (EI, 70 eV) *m*/*z* (%): 114 (<1) [M^+^], 99 (6), 70 (34), 55 (100), 42 (72), 39 (69); IR (ATR) 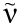: 2977 (w), 2935 (w), 2878 (w), 1762 (s), 1454 (w), 1387 (w), 1349 (w), 1293 (w), 1177 (s), 1121 (m), 1069 (m), 1042 (s), 994 (w), 949 (s), 922 (w), 872 (w), 775 (w), 704 (w), 625 (m), 569 (w) cm^−1^; UV–vis λ_max_ (log ε): 239 (1.22), 232 (0.69) nm. NMR spectroscopic data are in agreement with previously published data [33].

***cis*-2-Methylhexan-4-olide (9):** Yield: 0.19 g (1.41 mmol, 90%). TLC (hexane/ethyl acetate 3:1): *R*_f_ 0.36; GC (BPX-5): *I* = 1099; ^1^H NMR (400.1 MHz, CDCl_3_) δ 4.30–4.23 (m, 1H, CH), 2.65 (ddq, ^3^*J*_H,H_ = 8.5, 12.0, 7.0 Hz, 1H, CH), 2.47 (ddd, ^3^*J*_H,H_ = 5.4, 8.6 Hz, ^2^*J*_H,H_ = 12.4 Hz, 1H, CH_2_), 1.77 (dquin, ^2^*J*_H,H_ = 14.4 Hz, ^3^*J*_H,H_ = 7.2 Hz, 1H, CH_2_), 1.68–1.57 (m, 1H, CH_2_), 1.48 (dt, ^2^*J*_H,H_ = ^3^*J*_H,H_ = 12.2 Hz, ^3^*J*_H,H_ = 10.4 Hz, 1H, CH_2_), 1.25 (d, ^3^*J*_H,H_ = 7.1 Hz, ^1^*J*_C,H_ = 128.3 Hz, 3H, CH_3_), 0.98 (t, ^3^*J*_H,H_ = 7.5 Hz, ^3^*J*_H,H_ = 126.4, 3H, CH_3_); ^13^C NMR (100 MHz, CDCl_3_) δ (major compound) 179.6 (CO), 79.8 (CH), 36.8 (CH_2_), 35.9 (CH), 28.4 (CH_2_), 15.1 (CH_3_), 9.4 (CH_3_); MS (EI, 70 eV) *m*/*z* (%): 128 (<1) [M^+^], 127 (1), 99 (49), 84 (24), 71 (23), 69 (29), 56 (30), 55 (69), 41 (100), 39 (92); IR (ATR) 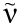: 3082 (w), 2973 (w), 2934 (w), 2883 (w), 1746 (s), 1659 (w), 1460 (w), 1383 (w), 1344 (w), 1281 (w), 1209 (w), 1087 (s), 1046 (w), 1023 (m), 1009 (m), 959 (m), 892 (m), 857 (m), 786 (m), 762 (w), 612 (w), 555 (w) cm^−1^; UV–vis: λ_max_ (log ε): 228 (1.44), 222 (0.90) nm.

**General procedure for the *cis*/*trans* isomerisation:** To a solution of the *cis*-substituted lactone **7** or **9** (1.0 equiv) in *t*-BuOH (0.2 M), KO*t*-Bu (2.0 equiv) was added. The mixture was stirred under reflux for 24 h and then quenched by the addition of an equal volume of HCl (0.5 M). The mixture was extracted three times with diethyl ether. The combined organic layers were dried over MgSO_4_ and concentrated under reduced pressure. Column chromatography of the residue on silica gel (pentane/diethyl ether 3:1) yielded mixtures of the *cis*- and *trans*-configured lactones as colourless oils, which were inseparable by chromatographic means.

***trans*-2-Methylpentan-4-olide (8):** Yield: 0.30 g (2.63 mmol, 59%, dr = 77:23, *cis*/*trans*), TLC (hexane/ethyl acetate 2:1): *R*_f_ 0.48; GC (BPX-5): GC: *I* = 1006; ^1^H NMR (400 MHz, CDCl_3_) δ 4.63 (ddq, ^3^*J*_H,H_ = 5.0, 7.0, 6.4 Hz, 1H, CH), 2.74–2.62 (m, 1H, CH), 2.08–1.96 (m, 2H, CH_2_), 1.33 (d, ^3^*J*_H,H_ = 6.4 Hz, 3H, CH_3_), 1.24 (d, ^3^*J*_H,H_ = 7.3 Hz, 3H, CH_3_); ^13^C NMR (100 MHz, CDCl_3_) δ 179.9 (CO), 74.5 (CH), 36.9 (CH_2_), 33.9 (CH), 20.9 (CH_3_), 15.6 (CH_3_); MS (EI, 70 eV) *m*/*z* (%): 114 (<1) [M^+^], 99 (8), 70 (38), 55 (100), 42 (80), 39 (75); IR (ATR) 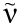: 2977 (w), 2936 (w), 2879 (w), 1763 (s), 1455 (w), 1386 (w), 1349 (w), 1178 (s), 1122 (m), 1068 (w), 1042 (m), 996 (w), 950 (s), 922 (w), 872 (w), 774 (w), 704 (w), 625 (w), 570 (w) cm^−1^; UV–vis λ_max_ (log ε): 239 (1.20), 232 (0.53) nm. NMR spectroscopic data are in agreement with previously published data, apart from the chemical shifts of the ^13^C NMR signals for the methyl groups, which were previously reported at 20.7 and 26.3 ppm [33], but found at 15.6 and 20.9 ppm in our spectrum.

***trans*-2-Methylhexan-4-olide (10):** Yield: 0.08 g (0.63 mmol, 71%, dr = 68:32, *cis*/*trans*). TLC (hexane/ethyl acetate 3:1): *R*_f_ 0.36; GC (BPX-5): *I* = 1105; ^1^H NMR (400 MHz, CDCl_3_) δ 4.43–4.36 (m, 1H, CH), 2.69–2.60 (m, 1H, CH), 2.12–2.04 (m, 1H, CH), 1.95 (dt, ^2^*J*_H,H_ = 12.9 Hz, ^3^*J*_H,H_ = 7.6 Hz, 1H, CH_2_), 1.74–1.50 (m, 2H, CH_2_), 1.26 (d, ^3^*J*_H,H_ = 7.2 Hz, 3H, CH_3_), 0.94 (t, ^3^*J*_H,H_ = 7.4 Hz, 3H, CH_3_); ^13^C NMR (100 MHz, CDCl_3_) δ 180.0 (CO), 79.6 (CH), 34.8 (CH_2_), 33.9 (CH), 28.2 (CH_2_), 15.8 (CH_3_), 9.5 (CH_3_); MS (EI, 70 eV) *m*/*z* (%): 128 (1) [M^+^], 99 (54), 84 (25), 71 (27), 69 (31), 55 (57), 41 (100), 39 (87); IR (ATR) 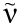: 2971 (w), 2983 (w), 2881 (w), 1762 (s), 1457 (w), 1378 (w), 1354 (w), 1291 (w), 1177 (s), 1134 (w), 1053 (w), 1024 (w), 996 (m), 960 (m), 929 (m), 868 (w), 755 (w), 733 (w), 583 (w) cm^−1^; UV–vis λ_max_ (log ε): 238 (1.30), 233 (0.92) nm.

**Enantioselective synthesis of (2*R*,4*S*)-7 and (2*S*,4*S*)-8.** Treatment of (2*S*,3*R*,4*R*)-**18** (15 mg, 0.06 mmol; its synthesis is published elsewhere [29]) with Et_3_N (12 mg, 0.12 mmol, 2 equiv) in dry CH_2_Cl_2_ (1 mL) for 1 h at room temperature was followed by acidic work-up with 2 N HCl (5 mL) and extraction with diethyl ether (3 × 5 mL). The combined organic layers were dried with MgSO_4_ and concentrated. The crude product was purified by chromatography over silica gel (hexane/EtOAc 3:1). Due to its volatility and the low amounts of material used for the synthesis, the solvent was not completely removed. The product (+)-**11** was identical to racemic **11** by GC–MS analysis (lit.: [α]_D_^25^ +91.5 (*c* 1.24, CHCl_3_), [34]). Compound **11** (6 mg) was dissolved in methanol (1 mL) and a small amount of Pd(OH)_2_ (ca. 1 mg, 10% Pd) was added. The catalytic hydrogenation was carried out in a H_2_ atmosphere (1 bar) at 20 °C for 24 h. The reaction mixture was filtered through silica gel and concentrated to yield (2*R*,4*S*)-**7**. The product was used in the next step without purification. Treatment of (2*R*,4*S*)-**7** with potassium *tert-*butoxide (5 mg) in *tert-*butanol (1 mL) under reflux for 6 h gave a mixture of (2*R*,4*S*)-**7** and (2*S*,4*S*)-**8**, which was used for enantioselective GC analyses.

**Agar diffusion assay for antimicrobial activity.** The substances were dissolved in MeOH at a concentration of 2 mg/mL. Twenty-five microlitres of the solutions (equal to 50 μg of the compounds) was pipetted onto a sterile filter disk (Schleicher & Schuell, 9 mm), which was placed onto an appropriate agar growth medium for the respective test organism and subsequently sprayed with a suspension of the test organism. The bacteria *Escherichia coli* and *Bacillus megaterium* were grown on Luria-Bertani (LB) medium (10 g L^−1^ peptone, 5 g L^−1^ yeast extract, 5 g L^−1^ NaCl, 20 g L^−1^ agar), the fungus *Microbotryum violaceum* and the alga *Chlorella fusca* were grown on MPY medium (20 g L^−1^ malt extract, 2.5 g L^−1^ peptone, 2.5 g L^−1^ yeast extract, 20 g L^−1^ agar), and *B. cinerea* was grown on biomalt agar (30 g L^−1^ biomalt, 20 g L^−1^ agar) [35]. Reference substances were penicillin, nystatin, actidione, and tetracycline, and negative controls were performed with MeOH alone. Commencing at the middle of the filter disk, the radius of the zone of inhibition was measured in millimeters.
